# Refinement and growth enhancement of Al_2_Cu phase during magnetic field assisting directional solidification of hypereutectic Al-Cu alloy

**DOI:** 10.1038/srep24585

**Published:** 2016-04-19

**Authors:** Jiang Wang, Sheng Yue, Yves Fautrelle, Peter D. Lee, Xi Li, Yunbo Zhong, Zhongming Ren

**Affiliations:** 1 State Key Laboratory of Advanced Special Steel, Shanghai University, Shanghai 200072, China; 2 The School of Materials, The University of Manchester, Oxoford Road, Manchester, M13 9PL, UK; 3 SIMAP/EPM 1130 rue de la Piscine BP 75 ENSEEG, 38402 St-Martin d’Heres Cedex, France

## Abstract

Understanding how the magnetic fields affect the formation of reinforced phase during solidification is crucial to tailor the structure and therefor the performance of metal matrix *in situ* composites. In this study, a hypereutectic Al-40 wt.%Cu alloy has been directionally solidified under various axial magnetic fields and the morphology of Al_2_Cu phase was quantified in 3D by means of high resolution synchrotron X-ray tomography. With rising magnetic fields, both increase of Al_2_Cu phase’s total volume and decrease of each column’s transverse section area were found. These results respectively indicate the growth enhancement and refinement of the primary Al_2_Cu phase in the magnetic field assisting directional solidification. The thermoelectric magnetic forces (TEMF) causing torque and dislocation multiplication in the faceted primary phases were thought dedicate to respectively the refinement and growth enhancement. To verify this, a real structure based 3D simulation of TEMF in Al_2_Cu column was carried out, and the dislocations in the Al_2_Cu phase obtained without and with a 10T high magnetic field were analysed by the transmission electron microscope.

Hypereutectic Al-Cu alloy is an ideal candidate for producing *in situ* composites because the multiphase structure that reinforced phases (*θ*-Al_2_Cu phase) embedded in a flexible matrix (Al-Al_2_Cu eutectic phase) can be directly achieved by casting^1^. How to optimize the microstructure of hypereutectic alloy attracts long-term attentions because the performance of composites can be further tailored by modifying multiphase’s microstructure such as the morphology of reinforced phases^2^. Implementing solidification control has been regarded as an efficient approach to achieve the microstructure optimization^3^, thus how to modify the solid structure of hypereutectic Al-Cu alloy via solidification control is worthy to study.

Applying magnetic field as an efficient solidification control method has been proposed more than half century ago^4^ and was firstly introduced to metallurgy with the purpose of damping the melt flow^5^. Over half century’s researches, the influence of magnetic field has been investigated on a wide range of metallic materials such as the pure metal^6^, single phase alloy^7^, monotectic alloy^8^ and the eutectic alloy^9^. These studies have uncovered several effects of magnetic field other than damping flows, for instance changing both the liquid-to-solid and solid-to-solid phase transition temperature^10^,^11^, redistributing the primary phase, inclusions and the solute^12^,^13^, and aligning the grain/crystal orientation^14^. However, little attention has been paid to its impacts on solidification of the near-eutectic alloys (hypo- or hyper-eutectic alloys), in particular on the formation of primary reinforced phases. Although a few works can be found^15^,^16^, including our previous ones^17^,^18^, these studies mostly focus on how the magnetic field affect the orientation of primary phase in near-eutectic alloys or the orientation relationship between two phases of the eutectic alloys.

Recently, a novel phenomenon that interaction between the thermoelectric currents and the applied magnetic field has been uncovered in the magnetic field assisting directional solidification of metals^19^,^20^. This interaction produces a kind of Lorentz forces that named thermoelectric magnetic forces (TEMF) because of its origins, and such forces exists in both solid and liquid phases^21^. TEMF in liquid can generate flow ahead of the solid/liquid interface^19^. This is contrary to the previous knowing that the uniform static magnetic field can only damp the melt flow, and thus drawn a huge of attentions^22^,^23^,^24^. Nevertheless, rare work has been done on understanding the influence of TEMF in the solid, and in particular its impact on the formation of primary reinforced phase during solidifying the metal matrix *in situ* composites.

Considering so, the hypereutectic Al-40 wt.%Cu alloys were directional solidified under different axial magnetic fields and the solidification structure was quantified in 3D using high resolution synchrotron X-ray tomography. The experimental results show that the primary Al_2_Cu phase was refined and its total volume was increased by the magnetic field. TEMF in the solid Al_2_Cu column was simulated based on its real structure, and the computed results indicate a torque can form on the column due to the TEMF. This torque would fragment the continuously grown column and respond to the refinement. In another aspect, TEMF causing dislocation multiplication can facilitate the atom attachment during faceted phase growth, which may lead to the Al_2_Cu phase’s total volume increase that we call growth enhancement in the present report.

## Results

**Figure 1:**
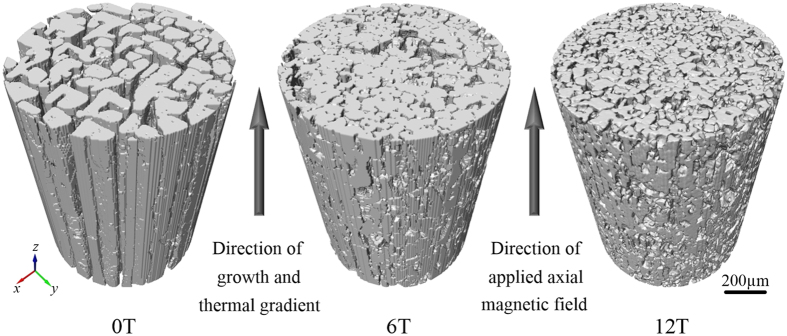
3D morphology of primary Al_2_Cu column solidified under different magnetic field flux intensities at the growth rate of 2 μm/s and thermal gradient of 6000 K/m.

Figure 1 shows the 3D morphology of primary Al_2_Cu columns achieved under axial magnetic fields of 0T, 6T and 12T respectively. The eutectic phases, which occupy the interspaces between columns, have been eliminated from the 3D view to expose the Al_2_Cu phase. Without magnetic field, many typical 90° angles between faces of the columns indicate that these Al_2_Cu phases are faceted, and it can see that these columns grow continuously in axial direction. The refinement of Al_2_Cu column can be observed both in transverse and longitudinal sections of the sample obtained under a 6T axial magnetic field, and the decrease of interspaces between columns means the increase of Al_2_Cu phase’s total volume. Moreover, less 90° angle faces in the columns suggests that the faceted feature of the primary Al_2_Cu phase tends to transform into a non-faceted appeal. By increasing the magnetic field to 12T, the degrees of refinement and growth enhancement further increase, and Al_2_Cu phases’ non-faceted feature become dominating.

**Figure 2:**
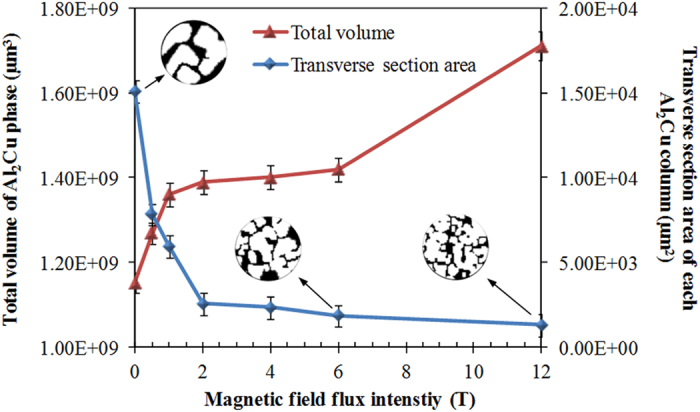
Total volume of the Al_2_Cu phase and transverse section area of each Al_2_Cu column plotted as a function of applied magnetic field flux intensities.

To confirm and quantify the refinement and growth enhancement of Al_2_Cu phase under magnetic field, the transverse section area of each column and their total volume were respectively calculated based on the 3D tomography data and plotted as a function of magnetic fields as shown in Fig. 2. It clearly shows that the total volume of primary Al_2_Cu phase increase with the magnetic fields, and reversely the transverse section area of each column gradually decrease. The decreased area indicates the refinement of the Al_2_Cu column. The 3D structure quantification shows that all the Al_2_Cu columns contacting with each other, which is understandable because columns may contact during growth or initially grow from the same seed. This makes identifying a single Al_2_Cu column difficult, and thus its transverse section area but not the volume was used to indicate the refinement effect. Better than cutting the sample into a number of slices and then measuring the transverse section area of columns in each slice to minimize the error, we can easily get sufficient slices using the tomography data. To more precise, the transverse section area of each Al_2_Cu column in Fig. 2 was produced by averaging that from 60 slices over the sample.

## Discussions

**Figure 3:**
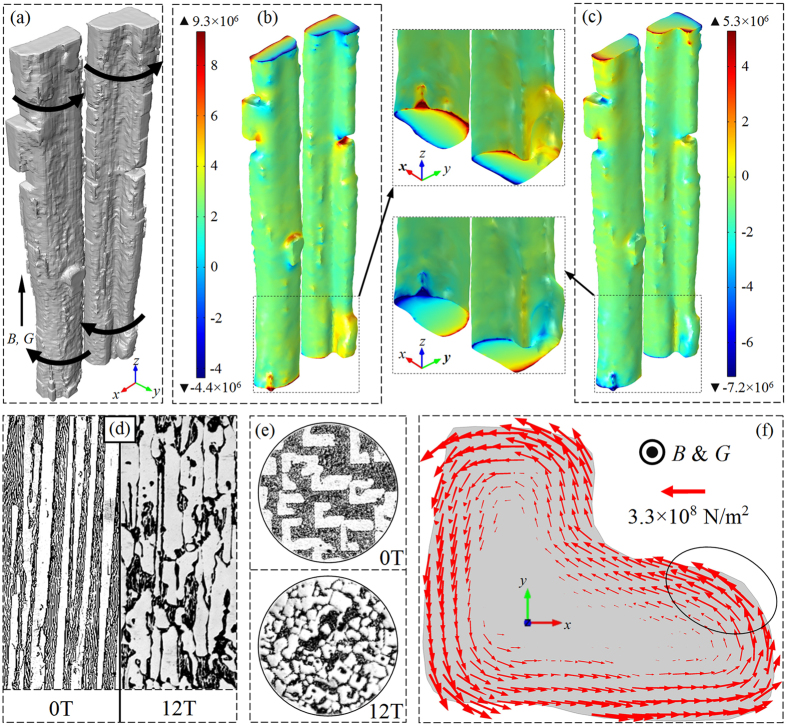
(**a**) Al_2_Cu column obtained without magnetic field; (**b**) x component magnitude of computed thermoelectric magnetic forces (TEMF) in Al_2_Cu column; (**c**) y component magnitude of computed TEMF in Al_2_Cu column; (**d**) and (**e**) Longitudinal and transverse structure of Al–40 wt.%Cu alloys obtained without and with magnetic field at growth rate of 2 μm/s; (**f**) Distribution of computed total stress in a transverse (x−y) plane at the column top. (For both experiment and simulation, B = 12T and G = 6000 K/m, the unit of colour legend is N/m^3^).

The refinement of Al_2_Cu column may attribute to its fragmentation^21^ caused by the TEMF because the present solidification conditions permit the interaction between thermoelectric currents and magnetic field. The thermal gradient along solid-liquid interface together with the dissimilar thermo-physical properties (e.g. thermoelectric power) between solid and the melt give rise to the occurring of Seebeck effect, which results in thermoelectric currents flowing through both solid and melt^25^. To confirm this and understand how TEMF fragment the Al_2_Cu column, a 3D simulation of TEMF was performed based on the real Al_2_Cu structure that got from the tomography data and shown in Fig. 3(a). The detailed description of simulation method can be found in ref. 26 and the related physical parameters of Al_2_Cu and the melt are listed in Table 1. The temperature field, electric current density and fluid flow field was coupled to simulate TEMF using a finite element method based commercial code COMSOL Multiphysics. Figure 3(b,c) respectively shows the *x* and *y* component magnitude of the computed TEMF in Al_2_Cu column under a 12T axial magnetic field. It can find that TEMF orientate anticlockwise at the top (hot region) and clockwise at the bottom (cool region), so that a torque as indicated by the black arrows in Fig. 3(a) forms on the column. As the Al_2_Cu always grows ahead the eutectic front, the excess part of Al_2_Cu column could be fractured by this torque. Considering so, the discontinuous growth of Al_2_Cu column in axial direction could form under magnetic field, and this is just the case indicated by Fig. 3(d) that the longitudinal structure of Al-40 wt.%Cu alloys fabricated without and with a 12T magnetic field. In fact, as reflected by Fig. 3(e), the decrease transverse section area of each column can be interpreted by the TEMF as well. Figure 3(f) is the distribution of computed total stress in Al_2_Cu column in a transverse (*x−y*) plane at the column top. Subjecting to such stresses this column should tend to rotate, as mentioned above this column may contact with another one at any point around its edge during the rotation. Assuming this column contacts with and is blocked by the other one at the point marked by the black circle, it would not be difficult to image that the upper left part of this column would depart away under the stresses orienting towards to negative *x* axis. It is worthy to point out that the transverse section area of each Al_2_Cu column decrease gradually with magnetic fields is because the TEMF is linearly proportional to the applied magnetic field flux intensity^21^.

**Figure 4:**
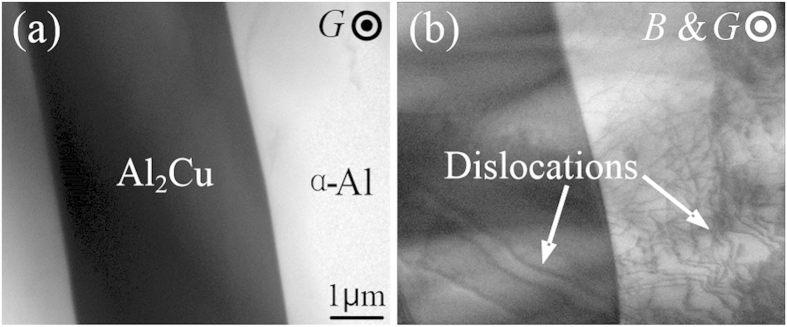
Bright field transmission electron microscope images of Al–40 wt.%Cu alloys obtained without (**a**) and with a 10T magnetic field (**b**) at the growth rate of 2 μm/s and the thermal gradient of 6000 K/m.

In terms of the increase of Al_2_Cu phases’ total volume, which is attributed to the fasted growth rate of the faceted phases when magnetic field is presence. It is known that the faceted phase’s growth rate is dominated by the atom attachment process, and the defects in the solid, like dislocations, could provide more vacancies for the approaching atoms to easy their locating. It is therefore not difficult to get that the dislocation multiplication could fast the growth of faceted phases. It is so happen that the stressed solid is a favourite condition for the dislocation multiplication, and thus the strong stresses (up to 3.3 × 10^8^ N/m^2^) caused by TEMF in the Al_2_Cu column are thought to be the main reason for the observed growth enhancement. To verify such interpretation, the dislocations in the solid were analysed by the transmission electron microscope. Figure 4 shows the bright field images of Al-40 wt.%Cu samples fabricated without and with a 10T magnetic field. It manifests that a number of dislocations form in the sample solidified under magnetic field and nearly dislocation free structure is obtained without the magnetic field. Moreover, it should point out that the continuous increase of Al_2_Cu columns’ total volume with magnetic field can be also explained by the linear proportion of TEMF to the applied magnetic fields.

Additionally, except the dislocation multiplication, we would like to mention several other phenomena those may contribute to the primary Al_2_Cu phases’ growth enhancement when the magnetic field is presence. In fact, the magnetic field induced change of phase transitions temperature^10^, and diffusion behaviour modification^27^ may also affect the growth of Al_2_Cu phase. Upon the change of phase transition, our previous work^28^ has been revealed that the magnetic field can increase the solidification undercooling of Al_2_Cu phase. The higher undercooling may lead to the faster growth rate and then increase the Al_2_Cu phases’ total volume. The altered diffusion behaviour may also contribute to the growth enhancement because the solidified Al_2_Cu phases continue to growth through diffusion.

## Conclusions

In summary, morphology of the primary Al_2_Cu phase obtained under different axial magnetic fields has been quantified for the first time in 3D using high resolution synchrotron X-ray tomography. Both refinement and growth enhancement of the Al_2_Cu were observed and their degrees increase with the applied magnetic fields. TEMF in solid Al_2_Cu column were confirmed by carrying out a real structure based 3D simulation, and capable to produce a torque on the column. The refinement is therefore attributed to the fragmentation of Al_2_Cu column by the torque. TEMF in solid Al_2_Cu leading to the dislocation multiplication was proposed, and verified by the transmission electron microscope analysis. This may respond to growth enhancement of the faceted Al_2_Cu phase, because defects in the solid can facilitate the atom attachment and thus fast the Al_2_Cu phase’s growth. The gradually increase degree of refinement and growth enhancement can be interpreted by the direct proportion between TEMF and the applied magnetic field flux intensity.

## Experimental Methods

The detailed experimental apparatus and process can be found in ref. 26. In brief, Al-40 wt.%Cu alloy was prepared with the high-purity Al and Cu elements by an argon gas (1 atm) filled induction furnace. The rod-like sample was 3 mm in diameter and 150 mm long, which was sealed in a corundum crucible with 3 mm inner diameter and 200 mm length. The directional solidification was upward and conducted by a Bridgman type furnace that was insulated from the Ga-In-Sn liquid metal pool by a refractory disc. The thermal gradient in the sample was controlled by adjusting the temperature of the furnace. The superconductor magnet supplied by Oxford Instrument Ltd. provided an axial magnetic field with adjustable intensity up to 14T. During the experiment, the sample within crucible was pulled down at a constant speed of 2 μm/s and an upward thermal gradient of 6000 K/m.

The high resolution synchrotron X-ray tomographic imaging of samples was carried out on Beamline I12 at the Diamond Light Source (DLS), Harwell, UK using a monochromatic beam of 53 KeV. The PCO.edge high resolution camera was used as the detector, and its pixel size is 1.3 × 1.3 μm^2^. The exposure time of 5 ms was chosen to guarantee the sufficient transmission and good contrast, the field of view is 3.3 × 2.8 mm^2^ (number of pixels of each X-ray image: 2560 × 2160 pixel), and 1800 frames were collected for one complete 3D tomography. Moreover, the detailed description of 3D tomography apparatus and reconstruction methods can be found in ref. 29. The volume data visualisation and quantification were carried out using Avizo 7 (Visualization Science Group, France) and Matlab (version 2012B with Image Processing Toolbox, Mathworks Inc., USA). The detail procedures have been described in ref 30, and the 3D renderings in this work were contoured at 40 cm^−1^.

## Additional Information

**How to cite this article**: Wang, J. *et al*. Refinement and growth enhancement of Al_2_Cu phase during magnetic field assisting directional solidification of hypereutectic Al-Cu alloy. *Sci. Rep*. **6**, 24585; doi: 10.1038/srep24585 (2016).

## Figures and Tables

**Table 1:** Physical parameters of Al_2_Cu phase and melt used for the 3D simulation.

Names and Units of the parameter	Al_2_Cu	Melt
Electrical conductivity, Ω^−1^·m^−1^	6.20 × 10^6^	3.05 × 10^6^
Absolute thermoelectric power, V·K^−1^	−0.60 × 10^–6^	−2.25 × 10^−6^
